# Remote influence of Atlantic multidecadal variability on Siberian warm season precipitation

**DOI:** 10.1038/srep16853

**Published:** 2015-11-23

**Authors:** Cheng Sun, Jianping Li, Sen Zhao

**Affiliations:** 1 College of Global Change and Earth System Science (GCESS), Beijing Normal University, Beijing 100875, China; 2 Joint Center for Global Change Studies, Beijing 100875, China; 3 State Key Laboratory of Numerical Modeling for Atmospheric Sciences and Geophysical Fluid Dynamics (LASG), Institute of Atmospheric Physics, Chinese Academy of Sciences, Beijing, China

## Abstract

The time series of 20th century Siberian warm season (May to October) precipitation (SWP) shows variations over decadal timescales, including a wetting trend since the 1970s. Here, it is shown that the Atlantic multidecadal variability (AMV) can be implicated as a remote driver of the decadal-scale variations in SWP. Observational analysis identifies a significant in-phase relationship between the AMV and SWP, and the SWP decadal variability can be largely explained by the AMV. The physical mechanism for this relationship is investigated using both observations and numerical simulations. The results suggest that North Atlantic sea surface temperature (SST) warming associated with the positive AMV phase can excite an eastward propagating wave train response across the entire Eurasian continent, which includes an east–west dipole structure over Siberia. The dipole then leads to anomalous southerly winds bringing moisture northward to Siberia; the precipitation increases correspondingly. The mechanism is further supported by linear barotropic modeling and Rossby wave ray tracing analysis.

Northern Hemisphere (NH) high-latitude precipitation is closely related to freshwater discharge into the Arctic Ocean^1^,^2^,^3^,^4^. Recent studies have reported sustained increases in river discharge from northern Eurasia into the Arctic Ocean that might affect global ocean circulation and climate^5^,^6^. In particular, rivers in Siberia (east of the Ural Mountains) contribute most of the total freshwater inflow to the Arctic Ocean^5^. For this reason, understanding the variability of Siberian precipitation is important for climate change research. Several studies have investigated the impacts of atmospheric circulation patterns on the variability of Siberian precipitation during the winter season^7^,^8^,^9^,^10^, in particular the Northern Annular Mode (NAM)^11^,^12^,^13^ and the Siberian High. In addition, sea surface temperatures (SSTs) in the Pacific and Atlantic oceans have been linked to changes in Siberian winter precipitation^14^,^15^,^16^.

The annual cycle of Siberian precipitation peaks during the warm season (May to October), and 60%–70% of the total annual precipitation in the three major Siberian river basins falls during this season^1^,^17^. This suggests that the hydrological cycle in Siberia is most active during the warm season. Therefore, it is reasonable to focus on the variability of warm season precipitation in this region and its possible causes. Previous studies have identified an east–west seesaw pattern as a marked characteristic of interannual variability of summer (June to August) precipitation in Siberia^17^,^18^,^19^. Another study found an anti-phase relationship between Siberia and northeast Asia in terms of interannual variations in July precipitation^20^. These studies have made good progress in understanding the interannual variability of Siberian warm season precipitation (SWP). However, limited efforts have been made to investigate the decadal-scale variations in SWP and its potential drivers. Several climate-modeling studies have highlighted the role of anthropogenic forcing in the intensification of the NH high-latitude hydrological cycle during the second half of the twentieth century^21^,^22^,^23^,^24^. Nevertheless, because of biases in the model representation of natural decadal climate variability^25^, the natural contribution to the observed change in the NH high-latitude hydrological cycle remains unclear.

The North Atlantic basin shows remarkable variability over decadal to multidecadal timescales, which has received a considerable amount of attention as it provides a large source of natural climate variability^26^,^27^,^28^,^29^. This variability is manifested by the oscillation of North Atlantic SST between basin-wide uniform warm and cold conditions, which has been commonly referred to as the Atlantic multidecadal variability (AMV)^30^,^31^. There is some consensus that the AMV is primarily driven by fluctuations in the strength of the Atlantic meridional overturning circulation (AMOC)^32^,^33^. Recent studies have suggested that an atmospheric circulation pattern, the North Atlantic oscillation (NAO), also plays an important role in the AMV^26^,^27^,^28^,^29^. The AMV is found to be closely related to climate variations over the Atlantic basin and adjacent continents^34^,^35^,^36^. However, to our knowledge, there has been no study linking the SWP to the AMV, and it is not known whether the AMV can influence Siberian precipitation over decadal timescales. This is not only important for understanding decadal climate variability over Siberia, but also has potential for decadal prediction in this region.

Herein, we employ multi-source atmospheric and oceanic datasets to explore the relation between the AMV and warm season precipitation over Siberia. The spatiotemporal characteristics of this relation are analyzed using correlation analysis and maximum covariance analysis (MCA; also known as singular value decomposition). The underlying mechanisms are investigated using an atmospheric general circulation model (AGCM) and Rossby wave theory.

## Results

**Figure 1:**
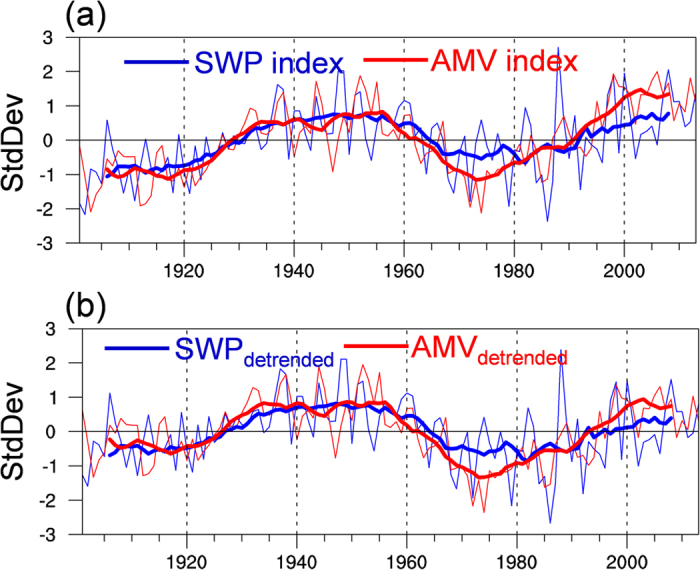
Observed temporal variations in SWP and AMV. (**a**) Time series of the SWP and AMV indices for the period 1901–2013 (thin line) and 11-yr running averages (thick lines), normalized by the long-term standard deviation. The definitions of the SWP and AMV indices are given in the text. (**b**) As in (**a**), but for the time series after removing the long-term linear trend. This figure was plotted using NCAR Command Language (NCL).

Figure 1 shows the normalized time series of AMV and SWP indices for the period of 1901–2013 derived from the Kaplan SST data and CRU precipitation data, respectively. The SWP index is defined as the area-weighted average of warm season precipitation anomalies over central Siberia (50°–70°N, 90°–125°E). As seen in Fig. 1a, the fluctuations in the AMV appear to be strongly in phase with those in SWP on decadal timescales. High correlation coefficients are observed between the AMV and SWP indices at zero lag based on both unfiltered (*r* = 0.59) and 11-yr running mean (*r* = 0.91) data for the period 1901–2013, both significant at the 95% confidence level. Moreover, significant simultaneous correlations are also found for other choices of running means (see Supplementary Figure 1). Because the raw AMV and SWP indices both show a weak positive trend for the period 1901–2013 (Fig. 1a), we re-examine the relationship based on the data after removal of the linear trend (Fig. 1b). The in-phase relationship is independent of the linear trend, and the correlation between detrended AMV and SWP remains strong (*r* = 0.52 for unsmoothed data, *r* = 0.86 for 11-yr running means, both significant at the 95% confidence level). Furthermore, the decadal variations of SWP (AMV) indices are consistent across different precipitation (SST) datasets (see Supplementary Figure 2), indicating that the results are not sensitive to the exact choice of dataset. Therefore, these results suggest that over decadal timescales, North Atlantic SST warming (cooling) is closely associated with wetter-than-normal (drier-than-normal) conditions over Siberia.

**Figure 2:**
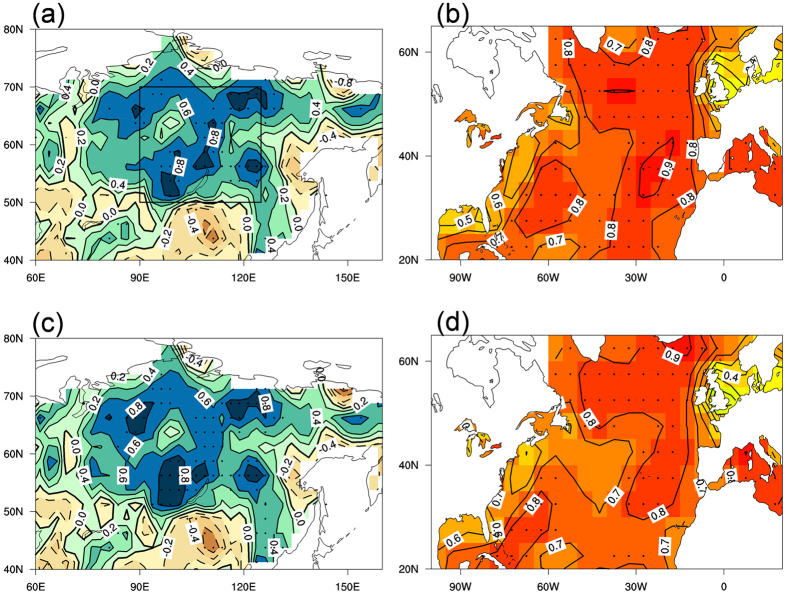
Spatial patterns of the decadal-scale relation between the SWP and AMV. (**a**) Correlation map between the warm season AMV index and land precipitation over northern Asia (40°–80°N, 60°–160°E) over decadal timescales for the period 1901–2013. The black box indicates the region used to define the SWP index. (**b**) As in (**a**), but for the SWP index and North Atlantic SST (20°–65°N). (**c**) Leading MCA mode for the warm season land precipitation over northern Asia over decadal timescales for the period 1901–2013, shown as a correlation map of the heterogeneous field with the MCA leading normalized expansion coefficients. (**d**) As in (**c**) but for North Atlantic SST. In (**a**,**b**) and (**c**,**d**), the long-term linear trends in SST and precipitation data were removed prior to the correlation analysis and the MCA, respectively. This figure was plotted using NCL.

The spatial patterns of the AMV–SWP relationship over decadal timescales are further analyzed. Figure 2a shows the correlation map between 11-yr running mean AMV index and warm season precipitation over northern Asia. Significant positive correlations are observed over large parts of the Siberian region, although the positive correlations are relatively small over some parts of the Yenisey River basin. The amplitude of the precipitation anomalies in association with the AMV is shown in Supplementary Figure 3 with significant positive values over the Siberian region and the regional average is about 1.6 mm month^−1^. On the other hand, the correlations between SST over the extratropical North Atlantic and the SWP index (Fig. 2b) exhibit a basin-wide uniform pattern with significant positive values that resembles the AMV. Furthermore, we apply the MCA^37^ to the cross-covariance matrix between warm season North Atlantic SST and northern Asian land precipitation (east of 60°E) over decadal timescales. The leading MCA mode explains 78% of the total squared covariance. The time series of the expansion coefficients associated with this mode are strongly correlated (*r* = 0.89, significant at the 95% confidence level). The expansion coefficients of the SST and precipitation fields have strong correlations of 0.98 and 0.96 with the smoothed AMV and SWP indices, respectively. Figure 2c,d shows the leading pair of heterogeneous patterns generated by correlating the respective heterogeneous field with the MCA leading normalized expansion coefficients. The precipitation pattern associated with the leading MCA mode bears a strong resemblance to the correlation map in Fig. 2a, with significant positive correlations over most of Siberia, while the SST pattern strongly projects onto the AMV. Thus, these results further suggest that the AMV–SWP relationship is strong and robust.

**Figure 3:**
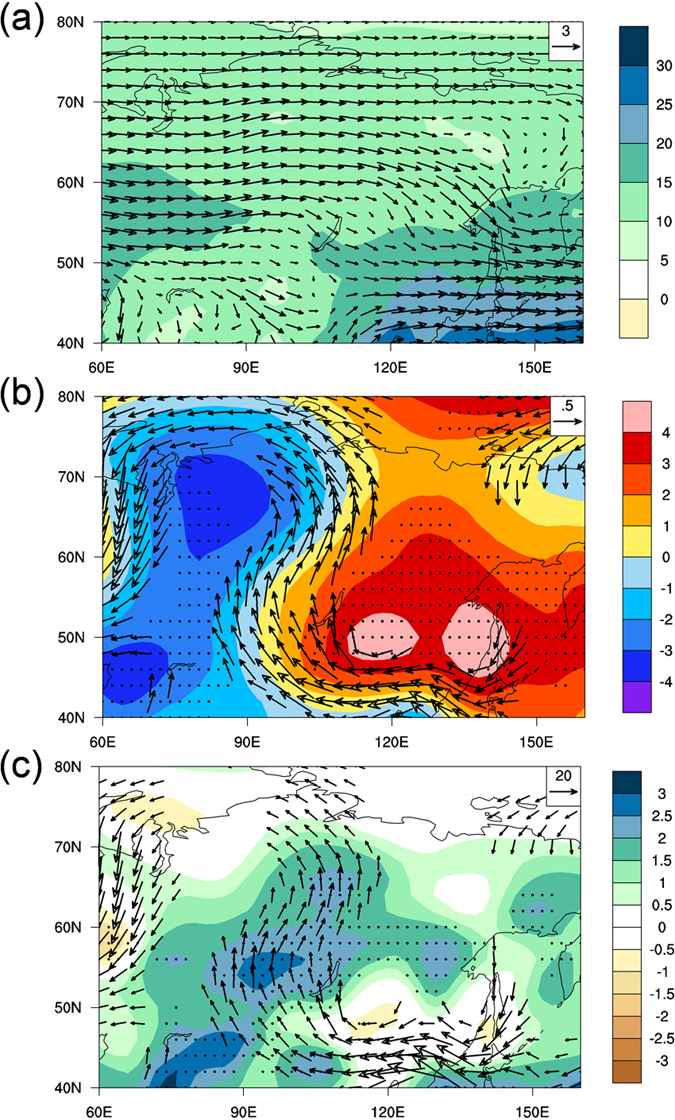
Large-scale atmospheric circulation pattern associated with SWP decadal fluctuations. (**a**) Long-term mean background states of warm season atmospheric precipitable water (shading; kg m^−2^) and low-level (700–850 hPa average) winds (vectors; m s^−1^) over northern Asia. (**b**) Composite differences in warm season low-level geopotential height (shading; m) and winds (vectors) over northern Asia between high- and low-SWP years. The high (low)-SWP years are identified if the decadal SWP is greater than one positive (negative) standard deviation of the index. The values in the dotted areas are significant at the 95% confidence level of the *t*-test for the geopotential height field; only the vectors significant at the 95% confidence level are shown. (**c**) As in (**b**) but for the vertically integrated moisture flux (vectors; kg m^−1^ s^−1^) and precipitable water fields (shading). This figure was plotted using NCL.

To understand the underlying mechanism of the AMV–SWP relationship, we first analyze the regional atmospheric circulation pattern that is favorable for precipitation over Siberia. Before examining precipitation variations, we will discuss averaged atmospheric properties in Siberia. As shown in Fig. 3a, during the warm season, the prevailing winds are westerly over Siberia. Long-term warm season mean atmospheric precipitable water shows an evident moisture contrast between inland and coastal regions, with wetter conditions along the Pacific coast. This gives rise to a southeastward moisture gradient over the southeast boundary of the Siberian region. Figure 3b presents the composite differences of low-level (700–850-hPa average) geopotential height and wind fields between high and low SWP years. The geopotential height field shows contrasting positive and negative anomalies in eastern and western Siberia, respectively, featuring an east–west dipole structure, and this leads to significant southerly anomalies of low-level winds between 90°E and 125°E. A simple geopotential height dipole index is therefore defined as the difference in area-averaged height between the positive-value box (45°–60°N, 110°–150°E) and the negative-value box (55°–70°N, 70°–90°E). As expected, the dipole index is highly correlated with the SWP index (*r* = 0.84, significant at the 95% confidence level) over decadal timescales.

The southerlies can result in northward moisture transport to Siberia and so increase the moisture supply there. To gain further insight into the moisture sources potentially associated with SWP decadal variability, we also examined moisture transport. Figure 3c shows the composite difference in vertically integrated (1000–400 hPa) moisture flux anomalies and the precipitable water between high and low SWP years. The vertically integrated moisture flux was calculated according to refs 38 and 29. Significant positive precipitable water anomalies accompanied by anticyclonic circulating moisture flux anomalies broadly cover most of Siberia. Anomalous northwestward moisture transport from the southern coastal region significantly contributes to the increased atmospheric precipitable water in inland Siberia, and reinforces the precipitation anomalies. Note that the pattern of moisture flux convergence (not shown) is similar to that of precipitable water. The anticyclonic moisture flux anomalies resemble the anomalous anticyclone shown in Fig. 3b, which is part of the geopotential height dipole. Therefore, the east–west dipole intensifies the moisture transport to Siberia, provides the moisture source for the positive anomalies of precipitable water, and consequently leads to the significant increases in precipitation.

**Figure 4:**
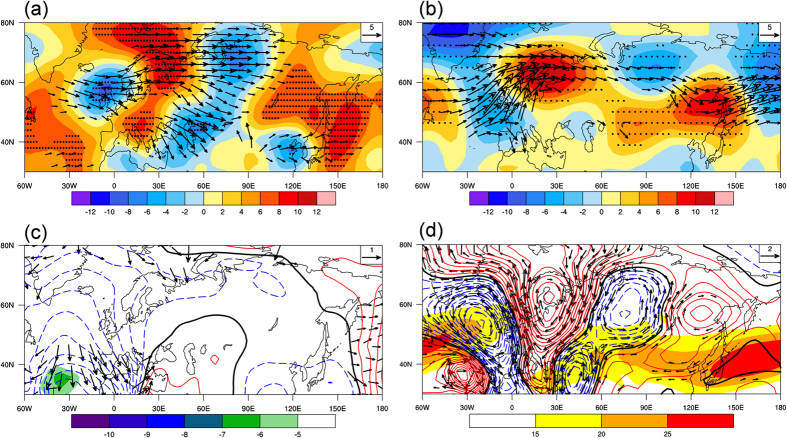
The downstream influence of AMV across the Eurasian continent. (**a**) Regression of the warm season NH 300-hPa geopotential height (m) with respect to the normalized AMV index at decadal time scales. Dots indicate regressions significant at the 95% confidence level. Vectors (m^2^ s^−2^, omitted below 1 m^2^ s^−2^) denote horizontal stationary wave activity flux associated with the wave train pattern. (**b**) Simulated warm season NH 300-hPa geopotential height (shading) and associated wave activity flux (vectors, omitted below 1 m^2^ s^−2^) in response to warm SST anomalies over the North Atlantic in the SPEEDY model. Dots indicate the regions where the results from the sensitivity simulations are significantly different from the control at the 95% confidence level for the height (Student’s t-test). (**c**) 300-hPa RWS (shading; 10^−11^ s^−2^, only absolute values larger than 5 × 10^−11^ s^−2^ are plotted), velocity potential (contours, interval is 10^5^ m^2^ s^−1^) and divergent wind (vectors; m s^−1^) anomalies associated with the AMV during the warm season, shown as regressions onto the normalized AMV index at decadal timescales. (**d**) Linear barotropic model response of 300-hPa geopotential height (contours, interval is 2 m) and associated wind fields (vectors) to the idealized vorticity forcing centered at 35°N, 40°W. The geopotential height was obtained by multiplying the streamfunction by the Coriolis parameter and the background color shading denotes the climatological mean of the warm season 300-hPa zonal wind. This figure was plotted using NCL.

In order to understand the role of the atmospheric circulation pattern in the AMV–SWP relationship, a regression map between the decadal-scale AMV index and 300-hPa geopotential height anomalies over the Eurasian continent is shown in Fig. 4a. A clear mid-latitude wave train with alternating positive and negative anomalies extends from the North Atlantic into the North Pacific across the Eurasian continent. The wave train has a typical wavelength of approximately 90–120° longitude, corresponding to a zonal wavenumber of about 3 to 4. The regression map of low-level field (900 hPa geopotential height) associated with the AMV index (Supplementary Figure 4) also shows good agreement with features in Fig. 4a, suggesting an equivalent barotropic structure of the wave train pattern. To better characterize this Eurasian wave pattern, an index is defined as the difference between the average height anomaly at two positive centers (65°N, 35°E and 50°N, 135°E) and the average height anomaly at two negative centers (55°N, 10°W and 65°N, 75°E). As expected, this wave train index is significantly correlated with the AMV over decadal timescales (*r* = 0.75, significant at the 95% confidence level). Moreover, it is evident that a significant pair of anomalous cyclonic and anticyclonic patterns over northern Asia, the downstream portion of this wave train, coincides with the Siberian geopotential height dipole shown in Fig. 3b that is closely associated with the SWP decadal variability. Moreover, the AMV index and the Siberian east–west dipole index are highly correlated over decadal timescales (*r* = 0.79, significant at the 95% confidence level). Thus, the zonal wave train over northern Eurasia links the SWP decadal variability to the AMV.

To further examine the relationship between North Atlantic SST forcing and the atmospheric circulation response over the Eurasian continent, we use the SPEEDY model to perform a sensitivity experiment. The control run was integrated for 15 years and forced with the climatological SST, and the last 10 years of the integration were used to provide the basic annual mean state. The sensitivity experiment was integrated for 12 years and forced with the AMV SST anomaly pattern (AMV-type SST anomaly composite only in the North Atlantic basin as shown in Supplementary Figure 5) imposed on the climatological SST. A 10-member ensemble mean was constructed from the last 10 years of the integration to reduce the uncertainties arising from different initial conditions. The atmospheric response to the SST forcing is defined as the difference between the ensemble means of the sensitivity and control runs. In addition, a longer 30-yr integration of the SPEEDY model, corresponding to a 30-member ensemble, was also performed to gain more confidence in the details of the responses, and the simulation results are similar (Supplementary Figure 7).

The simulated atmospheric circulation response to the AMV SST forcing during the warm season is shown in Fig. 4b. The simulated response also exhibits an eastward propagating wave train pattern across the entire Eurasian continent, resembling the zonal wave train shown in Fig. 4a. The simulated wave train also includes an east–west dipole over Siberia, although the dipole is slightly northwest–southeast oriented. This may be due to the difference between the real and simulated climatological mean basic states. We note that the model, forced by the North Atlantic SST warming, also reproduces the positive anomalies of precipitation and the associated anomalous southerly moisture flux over Siberia (Supplementary Figure 6). Moreover, the area averages of the simulated anomalies of precipitation and meridional moisture flux over the Siberian region are 1.8 mm month^−1^ and 19 kg m^−1^ s^−1^, respectively, comparable with the observed anomalies associated with the AMV (1.6 mm month^−1^ and 14 kg m^−1^ s^−1^). Thus, the model results suggest that the North Atlantic SST warming can excite a wave train propagating eastward to Siberia, leading to the anomalous northward moisture transport and consequent increase of precipitation there. As seen in Fig. 4b, the AMV can induce a significant teleconnection pattern across the entire Eurasian continent, but the most significant response of precipitation is found only in the Siberian region (Supplementary Figure 6). Precipitation is affected not only by the atmospheric circulation but also by the moisture source, and Siberia is close to the Pacific coast, which provides an important moisture source for the precipitation (as indicated in Fig. 3a). The AGCM-simulated 300-hPa geopotential height and precipitation anomalies over Eurasia in response to the cold AMV phase were also examined (Supplementary Figure 8) to see if the opposite effect could be produced. Compared with the AMV-warm-phase SST forcing experiment, the results show an almost reversed teleconnection pattern and decreased precipitation over Siberia responding to the cold-phase SST forcing. Therefore, these two experiments suggest that a significant response of SWP to the North Atlantic SST forcing may be found in both warm and cold phases of the AMV.

Previous studies have suggested that the NAO has a strong downstream impact on the East Asian climate through the subtropical jet stream waveguide and that the anomalous Rossby wave source (RWS) associated with the NAO plays an important role^39^,^40^,^41^,^42^,^43^. This study focuses on the downstream impact on Siberian climate of the AMV, which is different from the NAO. Figure 4c shows the anomalous velocity potential and RWS at 300 hPa in association with the AMV. The divergent winds and velocity potential indicate that anomalous upper-level divergence occurs in the warm AMV phase. A recent study has suggested that the upper-level divergent outflow associated with the warm AMV phase is consistent with the diabatic heating and ascending motion in response to the North Atlantic SST warming^44^. The anomalous RWS is calculated according to ref. 39. It shows a negative center located over the mid-latitude North Atlantic, in close correspondence to the anomalous upper-level divergence. This indicates that the anomalous RWS may originate directly from the divergence anomaly associated with the AMV. The AGCM simulated RWS and divergence in response to the warm AMV phase show generally similar features to those observed and are not shown here.

The steady Rossby wave response to the RWS shown in Fig. 4c is further examined using the linear barotropic model. Here, the warm season climatology (1961–1990) of the 300-hPa streamfunction is used as the basic flow. The steady response to the RWS forcing over the mid-latitude North Atlantic is characterized by an eastward propagating wave train from the forcing region to northern Asia (Fig. 4d). The similarity between the observed and simulated wave train patterns is evident. Moreover, the downstream portion of the simulated wave train also shows an east–west dipole and southerly wind anomalies over Siberia, consistent with the observations. Because the model is linear, the forcing associated with a convergence anomaly in the mid-latitude North Atlantic will produce a wave pattern completely out of phase with that in Fig. 4d. In addition, as the model is linearized about the basic state, the simulation results suggest that the warm season basic state may favor the eastward propagation of mid-latitude Rossby waves across the Eurasian continent. This is critical for understanding why the significant correlation between the AMV and Siberian climate occurs during the warm season. Rossby wave ray tracing theory 45,46 further allows us to characterize the pathway of the AMV’s downstream influence. The stationary Rossby wave ray is calculated following the methodology described in ref. 46. Rays of stationary waves with zonal wavenumber 3 and 4 from several initial points located within the North Atlantic RWS region are shown in Supplementary Figure 9. The warm season climatology of the 300-hPa zonal wind serves as the background field for the wave ray tracing. Zonal wavenumbers 3 and 4 are chosen to correspond to the spatial scale of the AMV-excited wave train pattern shown in Fig. 4a. The stationary wave rays for zonal wavenumbers 3 and 4 both show equatorward and eastward propagation. The wave rays propagate equatorward until they dissipate at critical latitudes in the tropics. The eastward propagating component extends across the entire Eurasian continent and reaches the Far East region, consistent with the pathway of the wave train response to the AMV. As seen in Supplementary Figure 9, the eastward wave trajectories over the North Atlantic region are generally embedded in the waveguide along the North Atlantic jet stream, while over the Eurasian continent, the trajectories are on the poleward side of the Asian jet stream and not overlapping with the jet stream. To address this, we further calculate the climatological stationary Rossby wavenumber (K_s_) for the warm season (Supplementary Figure 10). The calculation of K_s_ is based on the warm season basic zonal flow. The distribution of the warm season K_s_ suggests that stationary waves of lower wavenumbers (<5) are likely to propagate on the poleward side of the Asian jet stream, while smaller-scale waves with higher wavenumbers (≥5) tend to exist on the equatorward side. As mentioned above, the AMV-excited wave train to Siberia shows a structure at wavenumbers 3 to 4, and therefore tends to propagate on the poleward side of the jet stream.

## Discussion

Precipitation over the Siberian region plays a crucial role in the hydrological cycle in the Arctic domain and its variability has important implications for the Arctic climate. The present study presents observational evidence and numerical simulations to demonstrate that the Atlantic multidecadal variability (AMV), a dominant pattern of oceanic variability in the North Atlantic, could have a remote influence on the decadal variability of Siberian warm season (May to October) precipitation (SWP). Correlation analysis suggests a significant in-phase relationship between the AMV and SWP, and the SWP decadal variability can be largely explained by the AMV signal. The large-scale atmospheric circulation fields and moisture transport associated with SWP variability are further examined. The decadal variation in the SWP is strongly affected by an east–west geopotential height dipole pattern over northern Asia, which has a significant impact on the meridional moisture transport. Observational and AGCM modeling results suggest that the teleconnection pattern excited by North Atlantic SST warming shows an east–west dipole structure in Siberia leading to anomalous southerly wind and moisture flux, which favors the increase of SWP. Linear barotropic modeling and Rossby wave ray tracing results further demonstrate that the anomalous upper-level divergence over the mid-latitude North Atlantic occurring in the positive AMV phase can force a wave train propagating toward northern Asia. These results also highlight the role of the warm season basic flow in serving as a background field for the wave train, and therefore provide a theoretical explanation for the downstream influence of the AMV on the Siberian warm season climate.

Our findings provide strong evidence that during the past century the AMV has played an important role in modulating Siberian warm season climate on multidecadal timescales. It is known that the AMV has a period of about 50–70 years^47^,^34^,^28^. Thus, our results may have implications for predicting the climate of the next few decades. As shown in Fig. 4, the wave train pattern associated with the AMV extends to the North Pacific, implying a broader influence of the AMV on the downstream climate variability. More studies are needed to confirm this. In addition, the remote influence of the AMV on Siberian warm season climate has been demonstrated using a set of AGCM sensitivity experiments; further analysis of the downstream impact of the AMV within air–sea coupled models is also necessary.

## Methods

### Data

Two sets of monthly precipitation data for the global land surface were used in the present analysis: (1) the gridded precipitation dataset constructed by the Climate Research Unit (CRU TS 3.21)^48^ for the period 1901–2013, and (2) precipitation data from the Global Precipitation Climatology Centre full data reanalysis version 6.0 (GPCC v6)^49^, available for the period 1901–2010. The SST datasets (1901–2013) used in this study include the Kaplan SST dataset^50^ and the Hadley Centre SST dataset (HadSST3)^51^,^52^. Atmospheric data (1901–2012) were obtained from NOAA’s 20th Century Reanalysis^53^, and include wind fields, geopotential height and precipitable water (vertically integrated specific humidity). Warm season means are constructed for SST, precipitation and atmospheric data (e.g., winds, geopotential height) by averaging values from May to October. The observed AMV index is computed as the area-weighted average of SST anomalies over the extratropical North Atlantic region (30°–65°N, 75°–7.5°W) with respect to the 1961–1990 climatology^27^.

### Statistical Significance Test

The statistical significance of the linear regression coefficient and correlation between two autocorrelated time series is accessed via a two-tailed Student’s *t*-test using the effective number of degrees of freedom *N*^eff^ 54, which is given by the following approximation:


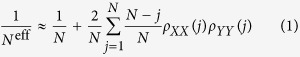


where *N* is the sample size and 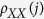 and 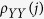 are the autocorrelations of two sampled time series *X* and *Y* at time lag *j*.

### Wave flux analysis

Wave activity analysis is applied to examine stationary Rossby wave energy propagation. The wave activity flux is parallel to the group velocity of stationary Rossby waves, and thus is a good indicator for the propagation direction and source of stationary Rossby waves in the atmosphere. The wave activity flux is computed following the method described in ref. 55.

### AGCM and linear barotropic model

Idealized numerical experiments were designed to investigate the atmospheric circulation response to a specific SST pattern. All experiments were carried out with the Simplified Parameterizations, primitivE-Equation DYnamics (SPEEDY) model developed at the Abdus Salam International Centre for Theoretical Physics^56^. SPEEDY is an intermediate complexity AGCM based on a spectral primitive-equation dynamical core. It is hydrostatic and uses sigma-coordinates and semi-implicit treatment of gravity waves. The resolution used here is triangular 30 (T30; i.e., ca. 3.75° × 3.75° horizontal resolution), with eight vertical levels. In this study, sensitivity experiments were conducted with SST anomalies imposed in the North Atlantic Ocean to isolate the impact of the AMV SST forcing on atmospheric circulation during the warm season. The spatial pattern and amplitude of the SST anomalies was derived by regressing warm season mean SST data onto a normalized version of the 11-yr running mean AMV index. In fact, the major feature of the AMV SST anomaly pattern, basin-wide warming/cooling in the North Atlantic as shown in Supplementary Figure 5, is essentially the same throughout the year. This SST anomaly pattern was then specified as a time-invariant, fixed forcing for the sensitivity experiments.

The linear barotropic model is a steady model with a horizontal resolution of T42 and consists of a simple barotropic vorticity equation given as:


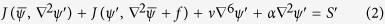


where *J* represents a Jacobian operator, 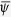 and 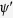 are basic state and perturbation streamfunctions, respectively, *f* is the Coriolis parameter, and 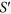 is the anomalous vorticity source induced by the divergence. The biharmonic diffusion coefficient 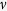 is chosen to be 2 × 10^16^ m^4^ s^–1^ in order to dampen small-scale eddies in one day, while the Rayleigh friction coefficient *α* is set to 10 day^–1^ 39,57.

### Graphics software

All maps and plots were produced using NCAR Command Language (NCL, ref. 58).

## Additional Information

**How to cite this article**: Sun, C. *et al.* Remote influence of Atlantic multidecadal variability on Siberian warm season precipitation. *Sci. Rep.* **5**, 16853; doi: 10.1038/srep16853 (2015).

## Supplementary Material

Supplementary Information
