# Supplementary material for: Remote influence of Atlantic multidecadal variability on Siberian warm season precipitation
Source: Sci Rep. 2015 Nov 23;5:16853. doi: 10.1038/srep16853 (PMC4655372; doi:10.1038/srep16853)
Supplement: Supplementary Information [file srep16853-s1.doc]

Supplementary Information for

**Remote influence of Atlantic multidecadal variability on Siberian warm season precipitation**

Cheng Sun, Jianping Li and Sen Zhao

*To whom correspondence should be addressed. E-mail: ljp@bnu.edu.cn

**This file includes:**

Supplementary Figs. 1–10

Supplementary Figure 1 Lead–lag correlation between the SWP and AMV indices for different smoothing time scales. The blue, red, and green lines are for unsmoothed, 5-yr, and 9-yr running mean time series, respectively. The definitions of the SWP and AMV indices are given in the text. The dashed lines are the 95% confidence levels based on the effective numbers of degrees of freedom. This figure was plotted using NCL.

Supplementary Figure 2 SWP and AMV indices from different SST and precipitation data sets. (a) Normalized time series of the SWP indices from the CRU and GPCC precipitation data sets (thin lines) and the 11-yr running averages (thick lines). (b) Normalized time series of the AMV indices from the Kaplan and HadISST3 SST data sets for the period 1901–2013 (thin lines) and the 11-yr running averages (thick lines). In (a) and (b) the long-term linear trends in SST and precipitation data have been removed to highlight the fluctuations. This figure was plotted using NCL.

Supplementary Figure 3 Regression of the warm season land precipitation anomalies over northern Asia (mm month^−1^) with respect to the normalized AMV index at decadal time scales. Dots indicate regressions significant at the 95% confidence level. This figure was plotted using NCL.

Supplementary Figure 4 Regression of the warm season NH 900-hPa geopotential height (m) with respect to the normalized AMV index at decadal time scales. Dots indicate regressions significant at the 95% confidence level. This figure was plotted using NCL.

Supplementary Figure 5 The AMV-type SST anomalies (K) used to force the sensitivity experiments in the SPEEDY model. This figure was plotted using NCL.

Supplementary Figure 6 Warm season precipitation (shading; mm month^−1^) and vertically integrated moisture flux (vectors; kg m^−1^ s^−1^) simulated by the SPEEDY model over the eastern NH in response to North Atlantic SST warming. Dots and arrows indicate the regions where the results from the sensitivity simulations are significantly different from the control at the 95% confidence level (Student’s t-test). This figure was plotted using NCL.

Supplementary Figure 7 SPEEDY model simulated fields from a longer 30-yr sensitivity experiment forced by the AMV-type warm SST anomalies. (a) 300-hPa geopotential height (shading; m), (b) precipitation (shading; mm month^−1^), and vertically integrated moisture flux (vectors; kg m^−1^ s^−1^) anomalies over the NH during the warm season. Dots in (a) and (b), and arrows in (b) indicate the regions where the results from the sensitivity simulations are significantly different from the control at the 95% confidence level (Student’s t-test). This figure was plotted using NCL.

Supplementary Figure 8 Simulated fields from the SPEEDY model of the warm season in response to North Atlantic SST cooling. (a) 300-hPa geopotential height (shading; m), (b) precipitation (shading; mm month^−1^), and vertically integrated moisture flux (vectors; kg m^−1^ s^−1^) anomalies over the NH. Dots in (a) and (b), and arrows in (b) indicate the regions where the results from the sensitivity simulations are significantly different from the control at the 95% confidence level (Student’s t-test). This figure was plotted using NCL.

Supplementary Figure 9 Stationary Rossby wave trajectories (blue curves) with zonal wave number (a) *k* = 3 and (b) *k* = 4. The Rossby waves start from the mid-latitude North Atlantic according to the RWS analyses shown in Fig. 4c, and are forced by the background state of the warm season climatology. The climatological mean 300-hPa zonal wind for the warm season (color shading; m s^–1^) serves as the background field for the Rossby wave trains. The trajectories are computed by Rossby wave ray tracing analysis, following ref. 46. This figure was plotted using NCL.

Supplementary Figure 10 Climatological Rossby stationary wavenumber ($\sqrt{(\beta-\overline{U}_{yy})/\overline{U}}$, where $\overline{U}$and $\beta$ are mean zonal wind and meridional gradient of planetary vorticity, respectively, and $-\overline{U}_{yy}$is the approximated meridional gradient of relative vorticity). The stationary Rossby wavenumber calculation is based on the long-term climatological zonal wind at 300 hPa for the warm season. This figure was plotted using NCL.
